# Transcriptome analysis of thermomorphogenesis in ovules and during early seed development in *Brassica napus*

**DOI:** 10.1186/s12864-023-09316-2

**Published:** 2023-05-04

**Authors:** Veronika Jedličková, Václav Hejret, Martin Demko, Pavel Jedlička, Marie Štefková, Hélène S. Robert

**Affiliations:** 1 Hormonal Crosstalk in Plant Development, Mendel Center for Plant Genomics and Proteomics, CEITEC MU—Central European Institute of Technology, Masaryk University, Brno, Czech Republic; 2 Bioinformatics Core Facility, CEITEC MU—Central European Institute of Technology, Masaryk University, Brno, Czech Republic; 3 Department of Plant Developmental Genetics, Institute of Biophysics of the Czech Academy of Sciences, Brno, Czech Republic

**Keywords:** *Brassica napus*, Thermomorphogenesis, Ovule, Seed, Embryo, Transcriptomics

## Abstract

**Background:**

Plant sexual reproduction is highly sensitive to elevated ambient temperatures, impacting seed development and production. We previously phenotyped this effect on three rapeseed cultivars (DH12075, Topas DH4079, and Westar). This work describes the transcriptional response associated with the phenotypic changes induced by heat stress during early seed development in *Brassica napus*.

**Results:**

We compared the differential transcriptional response in unfertilized ovules and seeds bearing embryos at 8-cell and globular developmental stages of the three cultivars exposed to high temperatures. We identified that all tissues and cultivars shared a common transcriptional response with the upregulation of genes linked to heat stress, protein folding and binding to heat shock proteins, and the downregulation of cell metabolism. The comparative analysis identified an enrichment for a response to reactive oxygen species (ROS) in the heat-tolerant cultivar Topas, correlating with the phenotypic changes. The highest heat-induced transcriptional response in Topas seeds was detected for genes encoding various peroxidases, temperature-induced lipocalin (TIL1), or protein SAG21/LEA5. On the contrary, the transcriptional response in the two heat-sensitive cultivars, DH12075 and Westar, was characterized by heat-induced cellular damages with the upregulation of genes involved in the photosynthesis and plant hormone signaling pathways. Particularly, the *TIFY/JAZ* genes involved in jasmonate signaling were induced by stress, specifically in ovules of heat-sensitive cultivars. Using a weighted gene co-expression network analysis (WGCNA), we identified key modules and hub genes involved in the heat stress response in studied tissues of either heat-tolerant or sensitive cultivars.

**Conclusions:**

Our transcriptional analysis complements a previous phenotyping analysis by characterizing the growth response to elevated temperatures during early seed development and reveals the molecular mechanisms underlying the phenotypic response. The results demonstrated that response to ROS, seed photosynthesis, and hormonal regulation might be the critical factors for stress tolerance in oilseed rape.

**Supplementary Information:**

The online version contains supplementary material available at 10.1186/s12864-023-09316-2.

## Background

Ambient temperature is a crucial environmental factor affecting plant growth and development. Temperate crops are sensitive to growth temperatures above a critical threshold of about 30 °C [1]. High temperatures have adverse effects on plant sexual reproduction, particularly on pollen development and pollen tube growth, as observed, for example, in tomato, rice, rapeseed, and Arabidopsis [2–5]. On the contrary, the impact of high-temperature stress on female gametophyte development is less known. It is considered more tolerant to heat than pollen [6]. Because of its inaccessibility due to being embedded within the maternal tissue, it has been sparsely studied in Arabidopsis [7] and a few crops [8]. We previously described the effects of elevated temperatures on seed development in *Brassica napus* [9]. Elevated temperatures result in substantial reductions in seed yield, consequent to defective ovule development, defective fertilization, and aborted seeds.

To dissect the developmental responses of the effects of elevated temperatures on seeds in flowering rapeseed plants, so-called seed thermomorphogenesis, three cultivars (DH12075, Topas DH4079, and Westar) were cultivated in diurnal growth conditions mimicking cool nights (18°C) and warm days (34°C) during the flowering period [9]. The long-term response to warm temperatures in such conditions may differ from acute heat stress with short high-temperature treatment at specific developmental stages. Our phenotyping analysis determined that Topas DH4019 was the most tolerant to heat stress for parameters including flowering time, number of produced flowers, apical dominance, and fertilization rate. Topas had the most aborted seeds (15%) at warm temperatures. However, embryonic development was less frequently defective in the viable seeds produced in Topas compared to those produced in the other two cultivars. On the other hand, DH12075 and Westar cultivars were more susceptible to elevated temperatures for the same analyzed features. Notably, seed abortion caused by heat stress was almost absent in DH12075 while having the highest frequency of defective embryos.

An embryo staging experiment uncovered that embryo development was faster at warm temperatures, meaning that embryos reached the same embryo stage on different days after pollination (DAP), depending on the growth temperature. In the control condition, the fertilized zygote elongates within 3 DAP. It undergoes a series of symmetric cell divisions to form an 8-celled embryo at 5-to-6 DAP. The following cell divisions are asymmetrical, giving rise to a globular embryo displaying signs of tissue specification (protoderm, lower tier, and upper tier) at 6-to-8 DAP. At 34°C, 8-celled embryos were identified at 4 DAP and globular embryos at 5 DAP. We, therefore, designed the experiments to compare gene expression patterns in seeds bearing embryos at the same developmental stage by collecting seeds at 5 DAP at 21°C and 4 DAP at 34°C for seeds bearing embryos at 8-cell stage (SE8) and 7 DAP at 21°C and 5 DAP at 34°C for seeds bearing embryos at globular stage (SEG).

We performed comparative transcriptome analyses of three cultivars (DH12075, Topas DH4079, Westar) in three tissues: unpollinated ovules, seeds bearing embryos at the 8-cell stage, and seeds containing globular embryos, under high-temperature growth conditions to complement our phenotyping analysis [9], aiming at revealing the molecular mechanisms underlying the thermomorphogenesis of embryo development in *B. napus*.

## Results

### Summary of the transcriptome sequencing dataset

RNA sequencing generated 1 097 million raw reads from 90 samples (three cultivars, three tissues, two temperatures, and five biological replicates). The raw reads in FASTQ format have been deposited to NCBI (BioProject accession number PRJNA885424). After filtering and trimming, 1 095 million high-quality clean reads were used for further analysis. The average Q20 and Q30 values were 91.5% and 88.7%, respectively (Additional file 1). We mapped the clean reads to the *B. napus* reference genome (Bra_napus_v2.0, GCF_000686985.2) with STAR v2.5.3a (average mapping rate of 87.4%) and quantified them using RSEM tool v1.3.1 [10].

### Identification of differentially expressed genes in the studied tissues and cultivars

The response to heat stress (differentially expressed genes, DEGs, 21 °C vs. 34 °C) was calculated for each tissue from each cultivar (Additional file 2, Fig. 1A). The number of up-regulated DEGs declines with the age of the samples: ovules > SE8 > SEG, except for Westar having a slightly higher number of up-regulated DEGs in SEG compared to SE8. In all samples but DH12075 SEG, there was a lower number of down-regulated DEGs than up-regulated ones for each sample. For example, only one-third of all DEGs are down-regulated in the ovules of Topas and DH12075. This analysis indicates that (1) ovules appear as the most heat-responsive organs from all three tested tissues; (2) the response is mainly transcriptional up-regulation. In addition, more genes were significantly regulated in the heat-sensitive cultivars (DH12075, Westar) than in the heat-tolerant Topas, especially in ovules.

**Fig. 1:**
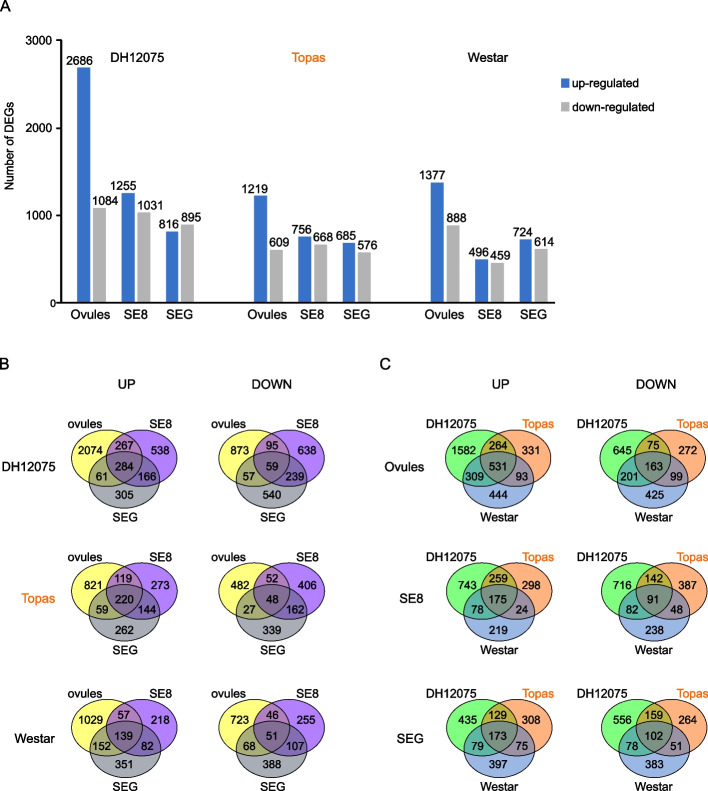
Transcriptional response of three rapeseed cultivars (DH12075, Topas, Westar) in ovules and developing seeds under heat stress. **A** Number of DEGs between control temperature and heat stress in all studied tissues and cultivars. **B**-**C** Venn diagrams of up-regulated and down-regulated DEGs under heat stress in ovules and seeds bearing embryos at the 8-cell stage (SE8) and seeds containing globular embryos (SEG). The heat-tolerant cultivar Topas is highlighted in orange

Venn diagrams compare all DEGs in tissues and cultivars (Fig. 1B and C). Sets of 284, 220, and 139 genes were up-regulated in all three tissues of DH12075, Topas, and Westar, respectively (Fig. 1B). In addition, 531, 175, and 173 genes were commonly up-regulated in all cultivars in ovules, SE8 and SEG, respectively (Fig. 1C).

### Heat stress response pathways among the common transcriptional response

Gene ontology (GO) terms and Kyoto Encyclopedia of Genes and Genomes (KEGG) pathway enrichment analyses were performed for DEGs of all samples (Additional Files 3 and 4). As expected, the up-regulated genes of most tissues in all cultivars were involved in response to heat, including GO terms “response to temperature stimulus” (GO:0009266), “response to heat” (GO:0009408), and “heat acclimation” (GO:0010286). Further, genes involved in “protein stabilization” (GO:0050821), “protein folding” (GO:0006457), and “cellular response to unfolded protein” (GO:0034620) were up-regulated in all samples. Accordingly, we identified GO terms related to Heat Shock Proteins (HSP) and their role as chaperone proteins, up-regulated in most (if not all) of the samples (Additional File 3). The KEGG analysis identified “Protein processing in endoplasmic reticulum” (bna04141) as the pathway commonly up-regulated in all samples, supporting the GO terms enrichment analysis on protein processing.

The stress response reduced cell metabolism in all three cultivars, which agrees with a study on heat-stressed 20 DAP *B. napus* seeds [11]. Among down-regulated genes, KEGG pathways enrichment analysis revealed: “biosynthesis of secondary metabolites” (bna01110) in all samples but Topas SEG and “metabolic pathways” (bna01100) in all samples but DH12075 ovules and Topas SEG. This is supported by GO term GO:0005975 “carbohydrate metabolic process” being significantly enriched in down-regulated genes in all samples, except Westar SE8 seeds.

### Specific transcriptional response in the heat-tolerant cultivar Topas

DEGs identified in the tolerant cultivar Topas are candidate genes involved in heat tolerance-related processes. We filtered the up-regulated DEGs specific for Topas (331, 298, and 308 genes for ovules, SE8, and SEG, respectively; Fig. 1C) and performed GO terms and KEGG pathway enrichment analyses with these datasets. The up-regulated DEGs present only in ovules, SE8, and SEG of Topas were not significantly enriched (adjusted *p*-value < 0.05) in any of the KEGG pathways. The top enriched GO terms were “cytidine to uridine editing” (GO:0016554) and “DNA damage checkpoint” (GO:0000077) in ovules, “amine metabolic process” (GO:0009308), and “response to heat” (GO:0009408) in SE8, and “lipid transport” (GO:0006869) and “cellular response to hypoxia” (GO:0071456) in SEG (Additional file 5). Among the other significant GO terms, we identified up-regulated DEGs in pathways of the heat response: “regulation of reactive oxygen species biosynthetic process” (GO:1903426) and “response to reactive oxygen species” (GO:0000302) in SE8 and “positive regulation of flavonol biosynthetic process” (GO:1900386), “chaperone-mediated protein complex assembly” (GO:0051131), and “protein stabilization” (GO:0050821) in SEG (Additional file 5, Fig. 2A and B). This result suggests that heat stress induces the production of ROS molecules, from which Topas seeds respond by producing antioxidant molecules such as flavonols and flavonoids. Those tissues also protect their proteins from the heat with protein chaperone activity.

**Fig. 2:**
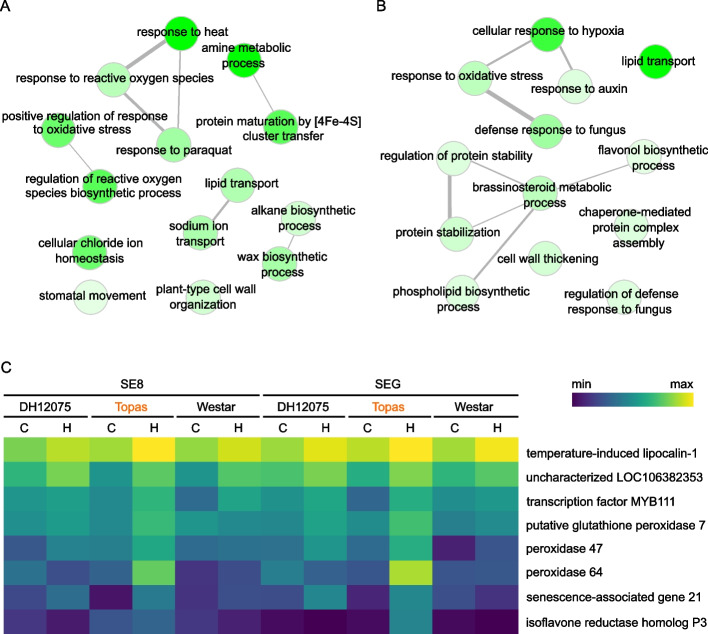
Specific response to the heat stress in seeds of tolerant cultivar Topas. **A**-**B** Gene ontology (GO) term enrichment analysis of DEGs in SE8 (**A**) and SEG (**B**) in the biological process category. Cytoscape networks were generated by REVIGO to reduce redundant GO terms. Color intensity represents the significance of enrichment (darker color = lower *p*-value). For details, see Additional file 5. **C** Transcriptional profiling of selected genes connected to reactive oxygen species (ROS) response and flavonol pathways. C, control conditions; H, high-temperature conditions

Transcriptional profiling of the selected DEGs connected to ROS and flavonol pathways is shown in Fig. 2C. Among the up-regulated DEGs showing the highest heat-induced response in SE8 and/or SEG of Topas, we identified genes coding for various peroxidases, TEMPERATURE-INDUCED LIPOCALIN-1 (TIL1) or flavonol-specific transcription activator MYB111. Interestingly, the list includes the uncharacterized *LOC106382353* gene, similar to *At1g13340* (coding regions of the genes share 83% nucleotide sequence identity), involved in response to oxidative stress in *Arabidopsis thaliana* [12]. This gene is up-regulated in SE8 of all cultivars, with Topas having the highest induction (log2 fold change of 1.73). In SEG, this gene is significantly up-regulated in Topas only (log2 fold change of 1.28). Similarly, *SENESCENCE-ASSOCIATED GENE 21/LATE EMBRYONIC ABUNDANT 5* (*SAG21*/*LEA5*) plays a specific protective role against oxidative stress by repressing photosynthesis [13, 14] and displays the highest heat-induced response in Topas seeds (log2 fold change of 4.15 and 3.55 in SE8 and SEG, respectively).

### Pathways activated in heat-sensitive cultivars

**Fig. 3:**
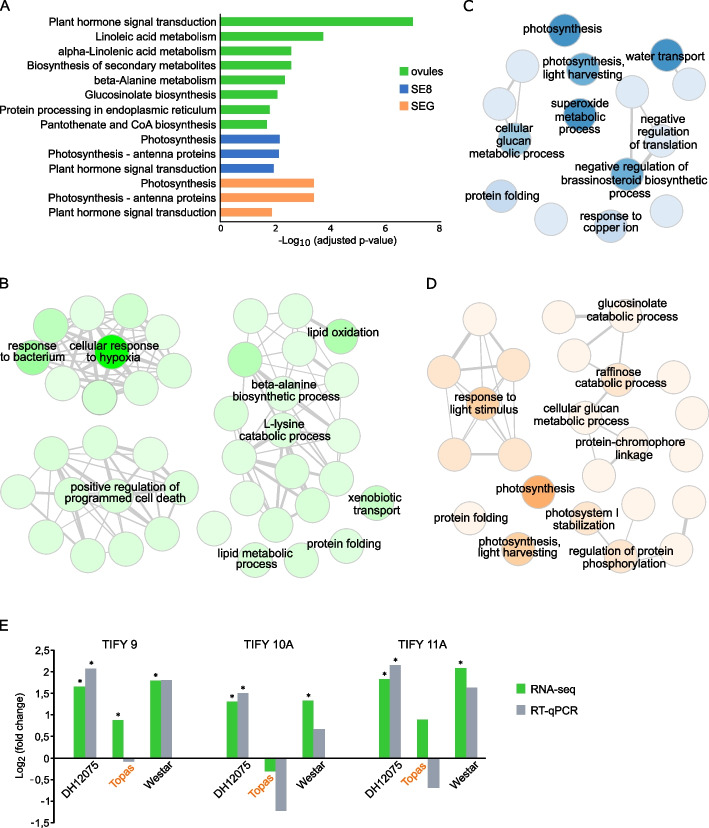
Specific heat stress response in ovules and seeds of sensitive cultivars DH12075 and Westar. **A** Significantly enriched KEGG pathways [20] in ovules (green), SE8 (blue), and SEG (orange). **B**-**D** Significantly enriched GO terms in the biological process category in ovules (**B**), SE8 (**C**), and SEG **D**. Cytoscape networks were generated by REVIGO to reduce redundant GO terms. The key terms are indicated in each network. Color intensity represents the significance of enrichment (darker color = lower *p*-value). For details, see Additional file 5. **E***TIFY* expression in ovules by RNA-seq data (green) and RT-qPCR (grey). The heat-tolerant cultivar (Topas) is highlighted in orange. *, significant difference (*p* < 0.05) between control and high temperature

The genes that are up-regulated specifically in sensitive cultivars DH12075 and Westar (while not induced in tolerant cultivar Topas) are related to stress-induced damage in plants contributing to the sensitivity of these cultivars. Thus, we extracted 2 335 DEGs in ovules (1 582 DEGs specifically up-regulated in DH12075 + 444 DEGs Westar-specific + 309 DEGs shared by these two cultivars), 1 040 DEGs in SE8, and 911 DEGs in SEG (Fig. 1C). The KEGG pathway enrichment analysis showed that DEGs in ovules are significantly enriched in bna04075 “Plant hormone signal transduction” and various metabolic pathways (Fig. 3A, Additional file 5). DEGs in SE8 and SEG were significantly enriched in “Photosynthesis” (bna00195), “Photosynthesis—antenna proteins” (bna00196), and “Plant hormone signal transduction” (bna04075). According to the GO terms analysis, the top-ranked enriched biological processes included the response to various stresses (e.g., hypoxia, water deprivation, stimulus from bacteria, heat) and metabolic processes in ovules, and photosynthesis-related terms in both SE8 and SEG (Fig. 3B, C, D, Additional file 5).

Among the genes classified into the “Plant hormone signal transduction” pathway, we identified several genes connected to auxin signaling. The genes encoding auxin-responsive proteins Aux/IAA16, Aux/IAA18, and Aux/IAA26 were up-regulated specifically in ovules of the sensitive cultivar(s) upon heat stress (Additional file 5). Aux/IAA proteins are transcriptional repressors of auxin response genes at low auxin concentrations [15]. Concurrently, the gene encoding the auxin influx protein AUX1-like protein 2 (LAX2) was down-regulated in the ovules of heat-sensitive cultivars, and *SUPPRESSOR OF MAX2 (MORE AXILLARY GROWTH 2) 1-LIKE 2 (SMXL2)* and *SMXL8* genes involved in the regulation of auxin transport [16–18], were down-regulated in heat-sensitive DH12075 ovules and Westar SE8, respectively (Additional file 2). These features of altered auxin distribution and signaling align with the detection of decreased IAA levels in heat-stressed Westar seeds. The same seeds displayed aberrant embryonic phenotypes similar to known mutants with altered auxin homeostasis [9].

Besides, the *TIFY/JAZ* (*JASMONATE ZIM/TIFY DOMAIN protein*) genes encoding transcriptional repressor proteins degraded upon the activation of the jasmonate signaling pathway [19] were over-represented among the DEGs in the hormone signaling pathway (bna04075) in ovules. The RNA-seq data were verified by RT-qPCR analysis and showed the same pattern of *TIFY9*, *10A*, and *11A* gene expression. The *TIFY* gene expression is induced by stress in heat-sensitive cultivars DH12075 and Westar and is not significantly affected in heat-tolerant Topas (Fig. 3E).

### Heat decreases plant metabolism

The down-regulated DEGs specific for heat-tolerant Topas (272, 387, and 264 genes for ovules, SE8 and SEG, respectively; Fig. 1C) were mainly enriched in KEGG pathways and GO terms connected to metabolic processes (e.g., starch, glycogen, glycosphingolipids, or glycine, serine, and threonine metabolism; Additional file 5). Down-regulated genes specific for heat-sensitive Westar and/or DH12075 (1 271 in ovules, 1 036 in SE8, 1 017 in SEG; Fig. 1C) were enriched in GO terms linked to cell cycle, cell division and cellularization in ovules, and sulfate metabolism in both SE8 and SEG (Additional file 5).

### Weighted gene co-expression network analysis

Weighted gene co-expression network analysis (WGCNA) was performed to describe the correlation patterns among expressed genes across all studied cultivars and tissues. We identified 24 modules (clusters) of highly correlated expressed genes (Additional file 6). The grey category is not a valid module; it contains genes that do not correlate well enough with one of the significant modules.

#### Analysis of significant temperature-responsive modules

**Fig. 4:**
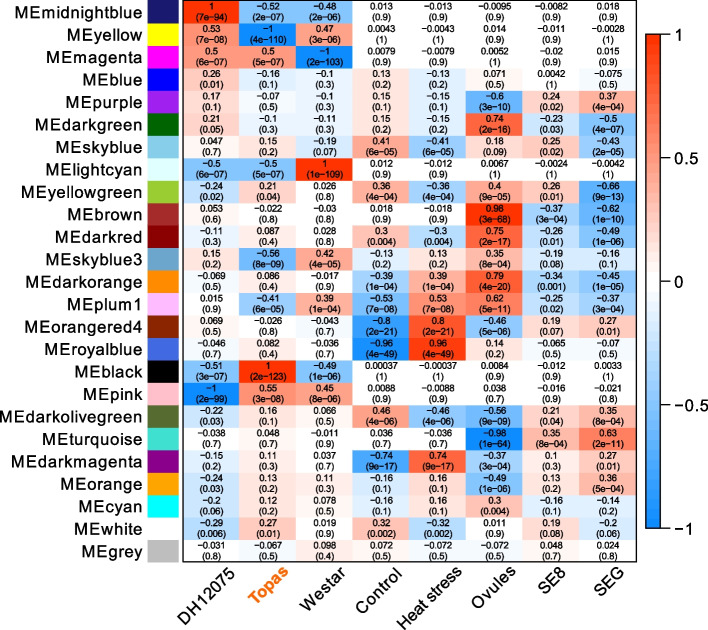
Module-trait relationship heatmap for different traits and gene modules provided by weighted gene co-expression network analysis (WGCNA). The value in the box indicates the correlation coefficient between the module and the trait, followed by the corresponding *p*-value (in brackets). The boxes are colored based on the correlation of the module with the trait: red is a strong positive correlation, while blue is a strong negative correlation. The heat-tolerant cultivar (Topas) is highlighted in orange

The co-expression network analysis identified ten modules that were significantly correlated (*p*-value < 0.05) with heat stress. Modules darkmagenta, darkorange, orangered4, plum1, and royalblue positively correlated with heat stress, while modules darkred, darkolivegreen, skyblue, white, and yellowgreen negatively correlated with treatment (Fig. 4, Table 1).

**Table 1:** Selected modules from WGCNA and their correlation with heat stress (H) or control conditions (C). G represents the significant correlation (positive or negative) with the tolerant genotype (Topas); such a module also displays the opposite correlation with sensitive cultivar(s). Significantly enriched KEGG pathways [20] and corresponding adjusted *p*-values are indicated at each module

Module	Correlation (correlation coefficient)	Significantly enriched KEGG pathways	adj *p*-value
Royalblue	H (cor = 0.96)	Protein processing in endoplasmic reticulum (bna04141) Spliceosome (bna03040)	5.56e-40 6.00e-06
Orangered4	H (cor = 0.8)	-	-
Darkmagenta	H (cor = 0.74)	Protein processing in endoplasmic reticulum (bna04141)	2.61e-06
Plum1	H (cor = 0.53) G (cor = -0.41)	Photosynthesis—antenna proteins (bna00196) Photosynthesis (bna00195)	7.70e-21 9.02e-20
Darkorange	H (cor = 0.39)	Plant hormone signal transduction (bna04075)	0.0212
Darkolivegreen	C (cor = 0.46)	Proteasome (bna03050)	6.73e-10
Skyblue	C (cor = 0.41)	Ribosome (bna03010)	1.15e-64
Yellowgreen	C (cor = 0.36) G (cor = 0.21)	Ribosome (bna03010)	1.12e-64
White	C (cor = 0.32) G (cor = 0.27)	Ribosome (bna03010)	2.18e-35
Darkred	C (cor = 0.3)	DNA replication (bna03030) Mismatch repair (bna03430) Ribosome biogenesis in eukaryotes (bna03008) Base excision repair (bna03410) Nucleotide excision repair (bna03420) Spliceosome (bna03040) Homologous recombination (bna03440) mRNA surveillance pathway (bna03015) Aminoacyl-tRNA biosynthesis (bna00970) RNA degradation (bna03018)	3.46e-05 4.06e-05 0.0003 0.0038 0.0075 0.0075 0.0103 0.0122 0.0171 0.0274
Black	G (cor = 1)	-	-
Yellow	G (cor = -1)	-	-
Skyblue3	G (cor = -0.56)	Photosynthesis—antenna proteins (bna00196) Other glycan degradation (bna00511)	0.0204 0.0322

KEGG pathways enrichment analysis (Table 1) showed that the genes in the module with the highest positive correlation coefficient with the heat stress (royalblue module, cor = 0.96) were significantly enriched (adjusted *p*-value < 0.05) in “Protein processing in endoplasmic reticulum” (bna04141) and “Spliceosome” (bna03040). On the other hand, the co-expressed genes in the darkolivegreen module, with the highest positive correlation coefficient with control conditions (cor = 0.46), were significantly enriched in “Proteasome” (bna03050). The top GO terms (Additional file 7) enriched in the royalblue module positively correlating with the heat stress were related to “response to heat” (GO:0009408), “protein folding” (GO:0006457), and “chaperone-mediated protein folding requiring cofactor” (GO:0051085), suggesting a co-expression of genes involved in protecting proteins from the heat stress. On the contrary, top GO terms associated with the darkolivegreen module of co-expressed genes negatively correlated with heat stress were “GDP-mannose metabolic process” (GO:0019673) and “proteolysis involved in cellular protein catabolic process” (GO:0051603). Royalblue and darkolivegreen modules represent the co-expressed gene clusters associated with the general response to heat stress, regardless of the cultivar.

The top 20 hub genes (i.e., genes with the highest module membership values, kME) of temperature-responsive modules are listed in Additional file 8. The top hub genes in the royalblue module mainly encode heat shock proteins. Top hub genes in darkolivegreen module encode proteins involved in various cellular processes such as ATP synthesis, protein trafficking, or cytoskeleton organization.

#### Co-expression modules associated with the heat-tolerant cultivar Topas

Our WGCNA identified ten modules with a significant positive or negative correlation with cultivar Topas (*p*-value < 0.05, Fig. 4). As Topas plants were more tolerant to warmer temperatures than the two other cultivars [9], the ten modules were screened for an opposite correlation coefficient in DH12075 and/or Westar. Six modules met this criterion. The yellow, skyblue3, and plum1 modules are negatively associated with Topas and positively with Westar and/or DH12075, while the black, yellowgreen, and white modules are positively associated with Topas and negatively with DH12075 and/or Westar (Fig. 4, Table 1).

Those modules were analyzed for KEGG pathways and GO terms enrichment (Table 1, Additional file 7). The co-expressed genes in the modules with the highest positive and negative correlation coefficient with Topas (black, cor = 1, and yellow, cor = – 1, respectively) were not significantly enriched in any of the KEGG pathways. Moreover, the genes of the black module did not have any significant enrichment (adjusted *p*-value < 0.05) in GO terms analysis, and the top 20 hub genes in this module encode mostly uncharacterized proteins (Additional file 8). We hypothesized that the set of these co-expressed genes may not be properly annotated or that the function of these genes does not differ from the entire dataset.

**Fig. 5:**
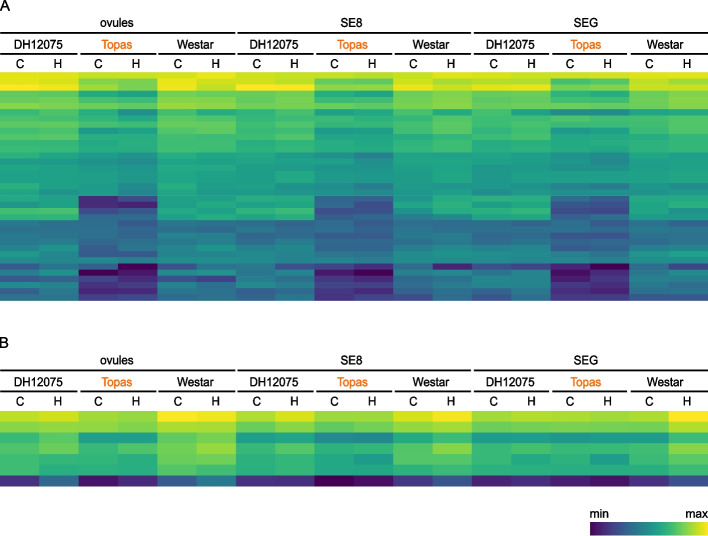
Transcriptional profiling of photosynthesis-related genes co-expressed in (**A**) yellow and (**B**) skyblue3 modules. Genes are associated with GO terms “photosynthesis” (GO:0015979) and “photosystem II” (GO:0009523) for the yellow module, and “photosynthesis” (GO:0015979) and “photosystem I” (GO:0009522) for skyblue3 module. The heat-tolerant cultivar Topas is highlighted in orange. **C**, control conditions; H, high-temperature conditions

The co-expressed genes in the yellow module are positively correlated with Westar and DH12075 and negatively correlated with Topas. Therefore, they may be involved in the negative regulation of heat tolerance. They are significantly enriched in the GO terms “photosynthesis” and “ceramide metabolic process” (biological process, GO:0015979, and GO:0006672, respectively) and “photosystem II” (cellular component, GO:0009523). The other module containing potential negative regulators associated with heat tolerance is the skyblue3 module (cor = – 0.56 with Topas and cor = 0.42 with Westar). The co-expressed genes were significantly enriched in “photosynthesis – antenna proteins” (bna00196) and “other glycan degradation” (bna00511) KEGG pathways (Table 1). In this module, we detected several significantly enriched GO terms connected to photosynthesis (GO:0015979, GO:0009765, GO:0018298, GO:0009522) (Additional file 7). In the top 20 hub genes of the skyblue3 module, we identified several genes associated with chloroplasts (chloroplastic UTP-glucose-1-phosphate uridylyl transferase 3, translocase of chloroplast 33, and ankyrin repeat domain-containing protein 2A). Transcriptional profiling of photosynthesis-related genes clustered in yellow and skyblue3 modules (Fig. 5) shows the overall low expression of these genes in tolerant cultivar Topas and higher expression in sensitive cultivars DH12075 and Westar.

#### Co-expression modules associated with heat stress response in tolerant rapeseed genotype

To better understand genotype-dependent heat stress tolerance mechanisms, we focused on the modules showing simultaneously (1) a significant heat stress response, and (2) a significant correlation with the heat-tolerant cultivar Topas, (3) while having an opposite correlation to heat-sensitive genotype(s). Three modules met this criterion: plum1, yellowgreen, and white (Fig. 4, Table 1).

The yellowgreen and white modules are negatively correlated with high-temperature conditions (cor = – 0.36 and cor = – 0.32, respectively), positively correlated with heat-tolerant cultivar Topas and negatively correlated with heat-sensitive cultivar DH12075 (Fig. 4, Table 1). GO terms and KEGG pathway enrichment analyses (Table 1, Additional file 7) revealed the genes connected to translation and ribosomes to be enriched in both modules. Top hub genes of yellowgreen and white modules code predominantly for ribosomal proteins (Additional file 8).

The plum1 module shows a positive correlation with heat stress (cor = 0.53), a negative correlation with the heat-tolerant cultivar Topas and an opposite (positive) correlation with the heat-sensitive cultivar Westar. This module is significantly over-represented in photosynthesis. KEGG pathways enrichment analysis revealed two pathways, “Photosynthesis—antenna proteins” (bna00196) and “Photosynthesis” (bna00195). The top enriched GO terms are “photosynthesis” (GO:0015979) and “photosynthesis, light harvesting” (GO:0009765) in the biological processes and “photosystem I” (GO:0009522) and “thylakoid” (GO:0009579) in the cellular components (Additional file 7).

**Fig. 6:**
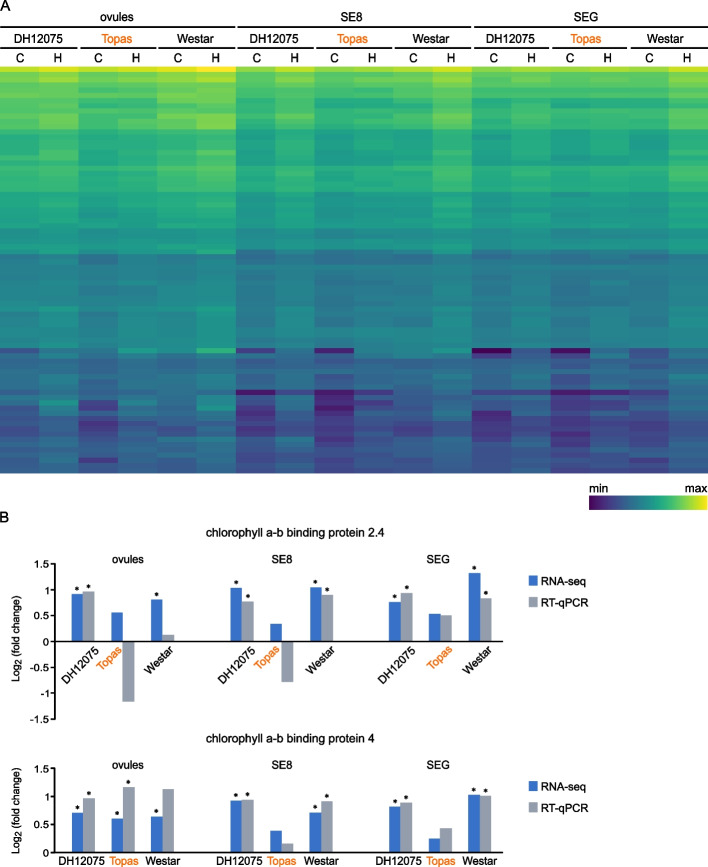
The expression profiles of the genes associated with the plum1 module.** A** Transcriptional profiling of all genes co-expressed in the plum1 module. **B** Expression by RNA-seq data (blue) and RT-qPCR analysis (grey) of two top hub genes of the plum1 module. The heat-tolerant cultivar Topas is highlighted in orange. *, significant difference (*p* < 0.05) between control and high temperature

Transcriptional profiling of all genes in the plum1 module was performed (Fig. 6A). The top hub genes of this module encode mainly proteins associated with photosynthesis (Additional file 8). The expression profiles of the top two hub genes of this module coding for chlorophyll a-b binding protein 2.4 and chlorophyll a-b binding protein 4 of light-harvesting complexes (LHC) are depicted in Fig. 6B, together with the verification by RT-qPCR analysis. The heat-sensitive cultivars DH12075 and Westar display the stress-induced up-regulation of the two genes in both SE8 and SEG. On the contrary, expression of these genes in Topas seeds is not significantly induced by heat stress.

## Discussion

The genetic and physiological impact of heat stress on different developmental stages of *B. napus* has been recently reviewed [21]. The transcriptome profile during *B. napus* seed and embryo development under normal growth conditions has been published [22–24], together with transcriptome responses to short-term heat stress in developing *B. napus* seeds [11]. Our previous phenotyping study of three cultivars of *B. napus* grown under long-term heat stress [9] revealed that Topas was more tolerant to high temperatures for most of the measured traits related to seed set and early seed development than the other two cultivars (DH12075 and Westar). To identify genes and pathways that may be involved in the higher tolerance of Topas compared to Westar and DH12075, we performed a comparative transcript profiling of ovules and young seeds from control and heat-stressed rapeseed plants of these three cultivars.

In all studied tissues and cultivars, we observed a general heat-stress response characterized by the induction of heat shock proteins (HSPs). These chaperone proteins prevent the thermal aggregation of substrate proteins and facilitate their subsequent refolding and reactivation [25]. Activation of this conserved heat-response mechanism was also identified by comparative transcriptome profiling of non-stressed and heat-stressed *B. napus* plants in various organs and tissues, namely leaves, roots, pistils, pollen, and siliques at the seed-filling stage [26–29]. Some HSPs are developmentally regulated, having specific functions during seed maturation and desiccation. In Arabidopsis, heat-stressed embryos showed an increase in HSP17.4 promoter activity and different spatial regulation of the promoter when compared to the non-stressed embryos, suggesting that the expression of some HSPs is regulated by distinct stress-mediated and developmental factors [30]. In our study, a small HSP, *BOBBER1*, was up-regulated in most tissues in *B. napus* (LOC106390591; Additional file 2)*.* In Arabidopsis, BOBBER 1 is required for thermotolerance in seedlings [31] and embryonic development [32]. Thus, it may be interesting to investigate the involvement of such small HSP in the seed thermoresponse.

Genes specifically up-regulated in the heat-tolerant Topas cultivar upon high-temperature treatment may play an essential role in heat stress tolerance. An analysis of the DEGs specific to Topas revealed the induction of genes connected to the reactive oxygen species (ROS) production and response. ROS are highly reactive reduced forms of atmospheric oxygen capable of oxidizing various cellular components leading to cellular oxidative damage. In plants, the cellular level of hydrogen peroxide, one of the reduced oxygen species, is mainly regulated by the enzymatic actions of catalases and peroxidases [33]. Peroxidases respond to environmental stresses, such as drought or salinity [34]. Tolerance to aluminum stress in transgenic tobacco plants was improved by overexpressing the Arabidopsis *AtPrx64* peroxidase [35]. Peroxidases are also responsive to heat stress. For example, peroxidase activities were induced in strawberry plants in response to heat shock and even more strongly in heat-acclimated plants undergoing gradual heat stress [36]. In our analysis, several peroxidases (peroxidase 47, peroxidase 64, glutathione peroxidase 7) showed the highest heat-induced transcriptional response in Topas seeds.

A similar expression pattern was also observed for the *TIL1* gene. Plant temperature-induced lipocalins are associated with tolerance to abiotic stresses, such as low or high temperature, oxidative stress, drought, and high light stress, probably through the promoted scavenging of ROS [37–41]. The absence of the Arabidopsis TIL1 induced severe defects in basal and acquired thermotolerance. However, *TIL1* over-expressing plants did not display significant thermotolerance enhancement [38]. Similarly, the Arabidopsis *til1* mutants were more susceptible to salt. But the heterologous expression of *TIL* from the salt-resistant poplar did not rescue growth defects induced by salinity stress in Arabidopsis plants [42]. Nevertheless, compared to the wild-type, *TIL* over-expression significantly protected the photosynthetic pigments, especially chlorophyll b, from salt exposure. TIL1 may protect chlorophyll b from degradation by preventing an excess of sodium and chloride accumulation in the chloroplasts, probably by salt-induced trafficking of TIL [42].

Another candidate gene for thermotolerance is *SAG21*/*LEA5,* having the highest induction in Topas seeds upon heat stress. LATE EMBRYOGENESIS ABUNDANT (LEA) proteins are a diverse family of hydrophilic proteins that are abundantly synthesized during the late maturation phase of seed development. LEA proteins are essential in protecting plant tissues against a wide range of abiotic stresses, particularly dehydration and cold stress [43]. SAG21/LEA5 protein improved oxidative stress tolerance when expressed in yeast. Arabidopsis plants ectopically expressing the *SAG21/LEA5* gene performed better for shoot and root growth under optimal conditions and oxidative stress [14]. Interestingly, over-expression of *SAG21/LEA5* in Arabidopsis and barley resulted in greater drought-induced inhibition of photosynthesis than in wild-type plants [13, 14]. Thus, SAG21/LEA5 may play a specific protective function against oxidative stress involving reduced photosynthesis.

Green seeds of oilseed rape contain chloroplasts with thylakoid structures and enzymes of the photosynthetic machinery. Photosynthesis herein plays a crucial role in the improved efficiency of oil accumulation [44–46]. After exposure to high temperatures, oil accumulation, photosynthetic and respiration rates, and the maximum quantum yield of photosystem II are negatively affected in the developing *B. napus* seeds. Moreover, heat stress reduces the contents of light-harvest pigments [11]. Comparative transcriptome analysis of the heat-stress response in 20 DAP *B. napus* seeds revealed the up-regulation of genes involved in the response to high light intensity and genes associated with chloroplasts and photoinhibition [11].

Photosynthesis as a critical factor impacting the *B. napus* seeds’ heat-stress response was also revealed in our analysis. We detected an overall higher expression of photosynthesis-related genes clustered in yellow and skyblue3 modules in sensitive cultivars DH12075 and Westar compared to their expression in the tolerant genotype Topas (Fig. 5), together with the heat-stress induction of specific genes connected to photosynthesis and antenna light-harvesting proteins in SE8 and SEG of heat-sensitive cultivars. In contrast, their expression remained unaffected in Topas seeds (Figs. 3 and 6). In Arabidopsis plants, enhanced expression of genes encoding photosynthetic proteins and several LHC antenna proteins correlated with impaired photosynthetic machinery upon the combination of high light and heat stress [47]. We can, therefore, speculate that the heat stress may damage the photosynthetic apparatus in seeds of the sensitive cultivars, and the proteins encoded by photosynthesis-related heat-induced genes might be involved in the renewal of damaged photosystem parts.

Impaired photosynthetic processes correlate with reduced oil accumulation in *B. napus* seed [11]. Oil production was reduced only by 2% in Topas seeds after the heat stress, while heat-sensitive cultivars DH12075 and Westar showed a 6% and 5% reduction, respectively [9]. Thus, the photosystems in Topas seeds might be less damaged, requiring less induction of photosynthetic genes. The potential higher protection of photosystems in Topas might be connected to the increased ROS response. Heat stress stimulates the over-production of ROS [48], which can cause damage to the photosynthetic apparatus [49], including LHC proteins [50]. Since we detected higher heat-induction of certain peroxidases in Topas SE8 and SEG (Fig. 2), this cultivar might exploit ROS-scavenging and quenching mechanisms to reduce damage to photosynthetic machinery in developing seeds.

Photosynthesis is also affected by the biosynthesis and signal transduction of some plant hormones [51]. Jasmonic acid (JA) is a plant hormone involved in many stress responses [19]. Notably, JA and salicylic acid confer a basal thermotolerance in Arabidopsis plants in response to acute heat stress [52]. JA is perceived by a nuclear SCF^COI1^ receptor complex. COI1 (CORONATINE INSENSITIVE1) is an F-box protein that targets the TIFY/JAZ proteins to degradation after its interaction with JA. The TIFY/JAZ proteins are negative regulators of the JA signaling, blocking JA-induced gene expression in the absence of JA. They do so by interacting with transcription factors; among them, some may be involved in the temperature stress response [19, 53]. The *TIFY/JAZ* gene expression is induced by JA, which contributes to fine-tuning JA signaling [54]. In our study, we observed the up-regulation of some *TIFY/JAZ* genes (Fig. 3) in the heat-sensitive cultivars, which could indicate an activation of the JA signaling pathway. It has been shown that JA plays a key role in the plant response to the combination of high light and heat stress [47, 55]. The increase of *TIFY/JAZ* and photosynthetic-related gene expression in the heat-sensitive cultivars may be an attempt to cope with the temperature stress. However, the molecular mechanisms remain elusive.

## Conclusions

To reveal mechanisms involved in thermomorphogenesis in ovules and young seeds in rapeseed, we compared transcriptome profiles of selected cultivars that differ in tolerance to long-term heat stress. We discovered a considerable number of DEGs specifically induced in heat-tolerant cultivar Topas that were connected to a response to oxidative stress. Besides, photosynthesis and plant hormone pathways, especially JA signaling, were shown to be important factors influencing the stress response in heat-sensitive cultivars DH12075 and Westar. Further examination can provide a more detailed understanding of interactions within the complex network of response to heat stress in seeds of the important oil crop *B. napus* and help to select the candidate genes for improving the seed development and hence seed yield and oil seed production under elevated temperatures.

## Methods

### Plant material, experimental conditions, and sample collection

Three *B. napus* spring cultivars (DH12075, Topas DH4079, and Westar) were cultivated as described previously [9]. Cultivar DH12075 is a double haploid line from a Cresor x Westar cross. DH4019 is a double haploid line selected from cultivar Topas. Briefly, bleach-sterilized seeds on MS plates were cold-stratified at 4 °C for 24 h and cultivated at 21 °C (16 h light / 8 h dark, 150 µmol/m^2^/s). Five-day-old plantlets were transferred to the soil. After two weeks, plants were fertilized with KRISTALON™ Start (N-P-K (19–6-20), 3% Mg, 7.5% S). With the first visible flower buds, the pots were transferred to the greenhouse chambers (Photon Systems Instruments, s.r.o.). The chambers were maintained with a 16 h light/8 h dark regime (150 µmol/m^2^/s light intensity, 35–45% humidity) and 18 °C during the night. During the day, control (CT) and high-temperature (HT) chambers were set to 21 °C and 34 °C, respectively, with ramping of the temperature up and down by 4 °C per hour. Watering was done manually in the trays to avoid any effect associated with drought stress. Plants were once fertilized with KRISTALON™ Fruit and Flower (N-P-K (15–5-30), 3% Mg, 5% S) at the flowering start. The samples were collected between October 2019 and March 2020. The collection of material was performed during specific hours of the day (12 – 3 p.m.) to reduce the circadian rhythms influence. Tissue was collected only from flowers in positions 5 to 65 on the main flowering stem (counted from the bottom) and 5 to 25 on the side flowering stem. For the collection of ovules, the flowers were emasculated one day before opening and harvested the day after. For the collection of seeds, flowers were pollinated on the day of opening and harvested 5 days after pollination (DAP) and 7 DAP in CT, 4 DAP and 5 DAP in HT to match the phases of embryo development in both temperature regimes (8-cell stage and globular stage). Samples (100 mg per biological replicate) were snap-frozen in liquid nitrogen and stored in a − 80 °C freezer. Five biological replicates were used for each treatment.

### RNA extraction, library construction, and sequencing

Total RNA from 100 mg of ovules or seeds was extracted using TRIzol reagent (Thermo Fisher Scientific) following the manufacturer’s protocol. RNA isolates were treated with rDNase (Macherey–Nagel) to remove traces of contaminant DNA, and the samples were subsequently purified using an RNeasy MinElute Cleanup Kit (Qiagen). RNA integrity was assessed with the Fragment Analyzer (AATI). RNA libraries were prepared using 500 ng of high-quality RNA (RQN values ≥ 8.5) with QuantSeq 3’ mRNA-Seq Library Prep Kit FWD for Illumina (Lexogen). We used unique molecular identifiers (UMI Second Strand Synthesis Mix, Lexogen) to identify PCR duplicates and a unique dual indexing strategy (i5 Unique Dual Indexing Add-on Kit for Illumina, Lexogen) to reduce sample index crosstalk. Libraries were sequenced with an Illumina sequencing platform (NextSeq 500), and 75-bp single-end reads were generated. Per-cycle base call (BCL) files were converted to fastq format using bcl2fastq v.2.20.0.422 Illumina software for base-calling. The raw reads in FASTQ format have been deposited to NCBI (BioProject accession number PRJNA885424).

### Sequence data analyses

Quality check of raw single-end fastq reads was carried out by FastQC v0.11.8, and a quality trimming was performed using Trimmomatic v0.36 [56]. The clean reads were mapped to the reference *B. napus* genome (Bra_napus_v2.0, GCF_000686985.2) using STAR v2.5.3a [57] and quantified using the RSEM tool v1.3.1 [10]. Bioconductor package DESeq2 v1.20.0 [58] was used to perform differential expression analysis. Differentially expressed genes (DEGs) with |log2 (fold change)| ˃ 1 and adjusted *p*-value < 0.05 were identified as significant DEGs. We used clusterProfiler v3.12.0 package to test the statistical enrichment of DEGs in The Kyoto Encyclopedia of Genes and Genomes (KEGG) pathways [20]. The adjusted *p*-value < 0.05 was used as the enrichment cut-off criterion.

For the gene ontology (GO) enrichment analysis, we populated GO terms in poorly annotated *B. napus* genome using a similarity search of annotated nucleotide sequences from the *Arabidopsis* genus and Brassiceae tribe taxa against extracted genes of *B. napus* genome (GCF_000686985.2). The result of the local alignment search was filtered by the percentage of identical matches ≥ 80, the percentage of query coverage per subject ≥ 80, the ratio of alignment length to query and subject length ≥ 0.8, and the expected value of alignment ≤ 0.01. The matched sequences were then cross-referenced to UniProtKB. The genes aligned to *Arabidopsis thaliana* (taxon 3702) nucleotide sequences were considered as the genes with the highest credibility of annotation. These data were combined with annotated *B. napus* genes in The Gene Ontology Annotation (GOA) Database v2020-02–21 [59]. Finally, the genes aligned with other species, and corresponding annotations were included. The GO enrichment analysis was performed using the clusterProfiler v3.12.0 package. The resulting *p*-values were corrected using the Benjamini–Hochberg procedure. For the graphical representation of data, REVIGO [60] was used to reduce redundant GO terms.

The weighted gene correlation network analysis (WGCNA) was performed using the R package WGCNA v1.69 [61]. We removed features with consistently low normalized counts (norm. count < 20 in more than 90% of the samples). A signed hybrid network (power β = 6) was generated from 43 301 genes. The dynamic tree-cutting algorithm with parameters deep split = 2 and cut height for merging modules = 0.2 detected 25 distinct gene modules. Eigengene-based connectivity (kME) and corresponding *p*-value were calculated for the 43 082 genes clustered into 24 modules. The other 219 genes were outliers (grey module).

### Quantitative reverse transcription PCR (RT–qPCR)

To validate the reliability of the RNA-seq analysis, RT-qPCR of selected genes was performed. The sequences of the primer pairs are listed in Additional file 9. Since *B. napus* is an allotetraploid species, the primers might also amplify the homeologous genes. The rapeseed *ACTIN7* gene (LOC106384924, LOC106441419) was used as the internal control. The cDNA synthesis was performed with 1.5 μg RNA using M-MLV Reverse Transcriptase (Promega). The PCR reaction was performed using the FastStart Essential DNA Green Master (Roche) on a Lightcycler 96 (Roche) at 95 °C for 10 min followed by 40 cycles of 95 °C for 10 s, 60 °C for 10 s and 72 °C for 26 s. The efficiency of each primer pair was assessed by constructing a standard curve through five serial dilutions. A final melt-curve step was included post-PCR to confirm the absence of any non-specific amplification. Each sample was analyzed in three biological replicates with three technical replicates. Relative gene expression was determined using the method previously described [62]. The expression levels were evaluated by Welch’s t-test.

## Supplementary Information

**Additional file 1: Supplementary Table S1.** Overview of the RNA sequencing data.

**Additional file 2: Supplementary Table S2.** DEGs (21°C vs. 34°C) with |log2 (fold change)| >1 and adjusted *p*-value < 0.05 in all studied cultivars and tissues. lfcSE, standard error of the log2FoldChange; baseMean, mean of normalized counts of all samples.

**Additional file 3: Supplementary Table S3.** GO terms enrichment analysis in DEGs. Significantly enriched terms (adjusted *p*-value < 0.05) are sorted according to the GO terms group (P, Biological Process; F, Molecular Function; C, Cellular Component).

**Additional file 4: Supplementary Table 4.** KEGG pathways [20] enrichment analysis in DEGs. Significantly enriched pathways (adjusted *p*-value < 0.05) are shown for each tissue and cultivar.

**Additional file 5: Supplementary Table S5.** GO terms and KEGG pathways [20] enrichment analysis of DEGs specific for Topas and for sensitive cultivars DH12075 and Westar.

**Additional file 6: Supplementary Table S6.** List of genes co-expressed in specific modules.

**Additional file 7: Supplementary Table S7.** GO terms enrichment analysis of genes co-expressed in a specific module according to WGCNA analysis. Significantly enriched terms (adjusted *p*-value < 0.05) are sorted according to the GO terms group (P, Biological Process; F, Molecular Function; C, Cellular Component).

**Additional file 8: Supplementary Table S8.** Top 20 hub genes in WGCNA modules.

**Additional file 9: Supplementary Table S9.** List of primers used for RT-qPCR.

## Data Availability

The dataset supporting the conclusions of this article is deposited to the NCBI repository (BioProject accession number PRJNA885424).

## Abbreviations

COI1: CORONATINE INSENSITIVE1
CT: Control-temperature chambers
DAP: Days after pollination
DEG: Differentially expressed genes
GO: Gene ontology
HSP: Heat Shock Proteins
HT: High-temperature chambers
JA: Jasmonic acid
KEGG: Kyoto Encyclopedia of Genes and Genomes
LAX2: AUX1-like protein 2
LHC: Light-harvesting complex
ROS: Reactive oxygen species
SAG21/LEA5: SENESCENCE-ASSOCIATED GENE21/LATE EMBRYONIC ABUNDANT5
SE8: Seeds bearing embryos at 8-cell stage
SEG: Seeds bearing embryo at the globular stage
SMAXL2: *SUPPRESSOR OF MAX2 (MORE AXILLARY GROWTH 2) 1-LIKE 2*
TIFY/JAZ: JASMONATE ZIM/TIFY DOMAIN proteins
TIL1: TEMPERATURE-INDUCED LIPOCALIN-1
WGCNA: Weighted gene co-expression network analysis
